# To Investigate the Risk of Herpes Zoster in Women With Endometriosis: A Taiwan National Population-Based Cohort Study

**DOI:** 10.3389/fmed.2021.584322

**Published:** 2021-09-08

**Authors:** Chao-Yu Hsu, Der-Shin Ke, Cheng-Li Lin, Chia-Hung Kao

**Affiliations:** ^1^Department of Medical Education, Ditmanson Medical Foundation, Chia-Yi Christian Hospital, Chia-Yi, Taiwan; ^2^Department of Optometry/Medical Imaging and Radiological Sciences, Central Taiwan University of Science and Technology, Taichung, Taiwan; ^3^Center for General Education, National Taichung University of Science and Technology, Taichung, Taiwan; ^4^Department of General Education, National Chin-Yi University of Technology, Taichung, Taiwan; ^5^Rural Generalist Program Japan, GENEPRO, Asahi, Japan; ^6^Management Office for Health Data, China Medical University Hospital, Taichung, Taiwan; ^7^College of Medicine, China Medical University, Taichung, Taiwan; ^8^Graduate Institute of Biomedical Sciences and School of Medicine, College of Medicine, China Medical University, Taichung, Taiwan; ^9^Department of Nuclear Medicine and PET Center, China Medical University Hospital, Taichung, Taiwan; ^10^Department of Bioinformatics and Medical Engineering, Asia University, Taichung, Taiwan; ^11^Center of Augmented Intelligence in Healthcare, China Medical University Hospital, Taichung, Taiwan

**Keywords:** endometriosis, herpes zoster, pelvic pain, depression, cohort study

## Abstract

**Background:** The objective of this study is to investigate the occurrence of herpes zoster (HZ) in patients with endometriosis.

**Methods:** This retrospective population-based cohort study was conducted using the Taiwan National Health Insurance Research Database. Between 2000 and 2012, women aged ≥20 years with newly diagnosed endometriosis were enrolled into the endometriosis group. Each patient with endometriosis was randomly matched to 4 controls according to age and index year. All the patients were traced from the index date to HZ diagnosis, loss to follow-up, death, or the end of December 2013.

**Results:** In total, 19,147 patients with newly diagnosed endometriosis and 76,588 participants without endometriosis were enrolled. The incidence of HZ was higher in endometriosis persons (5.36 per 1,000 person-years) than in matched controls (4.43 per 1,000 person-years) (*p* < 0.001). After adjustment for age and comorbidities, patients with endometriosis age ≤ 49 years (adjusted hazard ratio [aHR] = 1.17) (*p* < 0.001) and 50–64 years (aHR = 1.27) (*p* < 0.05) showed significantly higher risk of HZ than the corresponding controls. Among women without any comorbidities, patients with endometriosis were 1.22 times (*p* < 0.001) more likely to have HZ than those without endometriosis.

**Conclusion:** Taiwanese women with endometriosis may have a higher rate of HZ occurrence. Endometriosis seems to be a high burden for affected women. Therefore, we suggest that clinicians should be aware of HZ among women with endometriosis, although there may be ethnic differences.

## Introduction

Endometriosis is a condition in which endometrial tissue grows outside of the uterus. It often develops in the pelvic cavity, including ovaries, fallopian tubes, cul-de-sac and on or in the organs like bladder and colon. Endometriosis may lead to infertility, pelvic pain, or dyspareunia in women.

The prevalence of endometriosis is 10–15% among reproductive-age women (1). A cross-sectional survey of endometriosis in the United States found that its prevalence was 6.1%, and half of the affected population was diagnosed with endometriosis between the ages of 18 and 29 years (2). A meta-analysis investigating the influence of race on the prevalence of endometriosis found that black women were less likely to develop endometriosis than white women. Moreover, Asian women were 1.63 times more likely to develop endometriosis than white women (3).

Herpes zoster (HZ) develops with the reactivation of the varicella-zoster virus. It is characterized by painful dermatomal vesicular rashes. A study of HZ incidence based on the health information system of medical institutions found that the incidence of HZ was 8.8 per 1,000 person-years, and this trend increased with age. The highest rate was 30.5 per 1,000 person-years among the patients aged ≥ 80 years (4). Postherpetic neuralgia is a painful complication affecting to 30% of patients with HZ (5). Postherpetic neuralgia may occur after recovery from the acute stage, and can last from months to years.

Several diseases related to chronic pain (6–10) represent a burden in affected individuals, and are associated with HZ occurrence. Whether endometriosis and endometriosis-induced pelvic pain are stressful factors for affected women, and induce HZ. The objective of this study is to investigate the HZ risk among women with endometriosis.

## Methods

### Data Source

This was a retrospective population-based cohort study. The data were obtained from the National Health Insurance Research Database (NHIRD). The National Health Insurance (NHI) program was implemented in 1995 and covers almost 99% of the 23 million Taiwanese. We used the Longitudinal Health Insurance Database (LHID), which is a subset of the NHIRD and contains 1 million insured persons. Data on the medical services provided by the NHI program are collected by the NHI Administration and are collated into the NHIRD. This longitudinal database contains patients' demographic and administrative information, including sex, year of birth, residence region, dates of admission and discharge, prescription drugs, surgical procedures performed, and diagnostic codes. In the NHIRD, the International Classification of Disease, Ninth Revision, Clinical Modification (ICD-9-CM), and the Procedure Coding System are adopted to define the diagnostic and procedure codes, respectively. In compliance with the Personal Information Protection Act, individual identifiers are encrypted before information is released for research.

### Study Population

Between 2000 and 2012, women aged ≥ 20 years with newly diagnosed endometriosis were enrolled in this study. Endometriosis was identified based on the diagnostic code (ICD-9-CM code: 617). The diagnosis of endometriosis could be made by the significant signs and symptoms from the patient's medical history and physical examination, imaging findings or tissues confirmation through pathologic examination. Most HZ patients were diagnosed with appearance of painful herpetiform vesicles in a restricted dermatomal distribution. Newly diagnosed HZ (ICD-9-CM code: 053) was defined as at least one visit with inpatient claims data or two visits with outpatient claims data after the index date. The date of endometriosis diagnosis was considered as the index date. Each patient with endometriosis was randomly matched to 4 controls according to age and index year. All the patients were traced from the index date to the diagnosis of HZ, loss to follow-up, death, or the end of December, 2013.

The presence of HZ was the primary outcome of this study. The risk of the outcome may have influenced by comorbidities, including diabetes mellitus (ICD-9-CM code: 250), coronary artery disease (CAD, ICD-9-CM codes: 410 - 414), depression (ICD-9-CM codes: 296.2, 296.3, 300.4, and 311), chronic kidney disease (CKD, ICD-9-CM codes: 585 and 586), obesity (ICD-9-CM code: 278), and cancer (ICD-9-CM codes: 140 - 208), which were recorded as covariates.

### Statistical Analysis

Student's *t*-test and the chi-square test were used to compare the difference between the endometriosis and control groups. The incidence of HZ (per 1,000 person-years) was analyzed in both groups through stratification by age and comorbidities. HZ occurrence was compared between the endometriosis and control groups and was analyzed using Cox proportional hazard regression models. We set the significance threshold at α = 0.05 for priori hypotheses. The *p* < 0.01 was considered statistically significant. All statistical analyses were performed using SAS statistical software (Version 9.4 for Windows; SAS Institute, Inc., Cary, NC, USA).

### Ethics Approval and Consent to Participate

The NHIRD encrypts patients' information to protect privacy and provides researchers with anonymous identification numbers associated with relevant information including sex, date of birth, medical services received, and prescriptions. Therefore, patient's consent is not required to access the NHIRD. This study was approved to fulfill the condition for exemption by the Institutional Review Board (IRB) of China Medical University (CMUH104-REC2-115-CR4).

## Results

**Figure 1:**
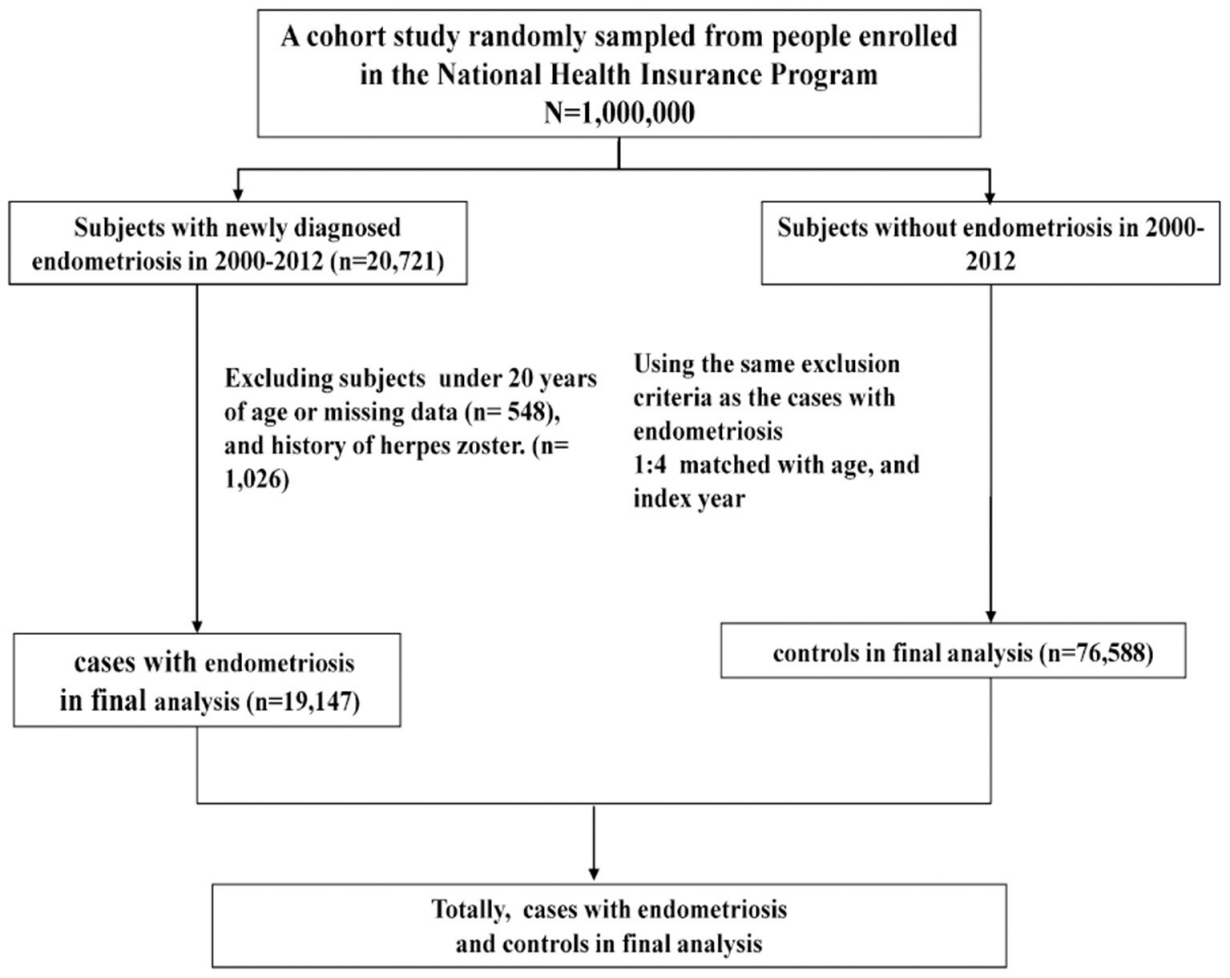
The procedure of selecting patients.

A total of 19,147 patients with newly diagnosed endometriosis and 76,588 participants without endometriosis were enrolled (Figure 1). No significant differences were observed between the endometriosis and control groups in age distribution. The mean age was 38.0 (±9.57) years for the endometriosis group and 38.4 (±8.88) years for the control group (Table 1). The majority of patients were aged ≤ 49 years. The mean follow-up time was 7.44 (±3.82) years for the endometriosis group and 7.42 (±3.83) years for the control group. However, several demographic data, such as diabetes mellitus, CAD, depression, obesity, and cancer, were significantly more frequent in the endometriosis group than in the control group. Only CKD was less prevalent in the endometriosis group than in the control group.

**Table 1:** Demographic characteristics and comorbidities in cohorts with and without endometriosis.

**Variable**	**Endometriosis**		***p*-value**
	**No**	**Yes**	
	***N* = 76,588**	***N* = 19,147**	
Age, year			0.99
≤ 49	70,972 (92.7)	17,743 (92.7)	
50–64	5,204 (6.79)	1,301 (6.79)	
65+	412 (0.54)	103 (0.54)	
Mean ± SD^†^	38.0 (9.57)	38.4 (8.88)	<0.001
**Comorbidity**			
Diabetes	991 (1.29)	323 (1.69)	<0.001
CAD	2,352 (3.07)	830 (4.33)	<0.001
Depression	2,961 (3.87)	1,072 (5.60)	<0.001
Chronic kidney disease	273 (0.36)	61 (0.32)	0.43
Obesity	1,292 (1.69)	427 (2.23)	<0.001
Cancer	813 (1.06)	243 (1.27)	0.01

*Chi-Square Test; ^†^: T-Test*.

*CAD, coronary artery disease*.

The incidence of HZ was higher in endometriosis persons (5.36 per 1,000 person-years) than in matched controls (4.43 per 1,000 person-years) (*p* < 0.001) (Table 2). The results revealed that patients with endometriosis were 1.20 times (95% confidence interval [CI] = 1.11–1.31; *p* < 0.001) more likely to have HZ than those without endometriosis. HZ occurrence was 2.53 times and 3.46 times higher among patients aged 50–64 and >65 years compared with those aged ≤ 49 years (95% CI = 2.29–2.80 and 2.65–4.51; *p* < 0.001). Older age or age more than 65 years, diabetes mellitus, CAD, depression, and cancer were significantly positively associated with HZ. Among the comorbidities, a significantly increased HZ risk was found in patients with diabetes mellitus (adjusted hazard ratio [aHR] = 1.41, 95% CI = 1.14–1.74; *p* < 0.01), CAD (aHR = 1.47, 95% CI = 1.28–1.70; *p* < 0.001), depression (aHR = 1.45, 95% CI = 1.24–1.69; *p* < 0.001), and cancer (aHR = 1.54, 95% CI = 1.18–2.00; *p* < 0.01). However, the association of HZ with CKD and obesity was non-significant (Table 2).

**Table 2:** The incidence and risk factors for herpes zoster.

**Variable**	**Event**	**PY**	**Rate^**#**^**	**Crude HR(95% CI)**	**Adjusted HR^**&**^ (95% CI)**
**Endometriosis**					
No	2,517	568,343	4.43	1.00	1.00
Yes	764	142,470	5.36	1.21 (1.12, 1.31)***	1.20 (1.11, 1.31)***
**Age, year**					
≤ 49	2,734	663,910	4.12	1.00	1.00
50–64	486	43,638	11.1	2.79 (2.53, 3.07)***	2.53 (2.29, 2.80)***
65+	61	3,266	18.7	4.65 (3.60, 5.99)***	3.46 (2.65, 4.51)***
**Comorbidity**					
**Diabetes**					
No	3,182	701,584	4.54	1.00	1.00
Yes	99	9,230	10.7	2.41 (1.97, 2.94)***	1.41 (1.14, 1.74)**
**CAD**					
No	3,042	687,181	4.43	1.00	1.00
Yes	239	23,633	10.1	2.29 (2.01, 2.62)***	1.47 (1.28, 1.70)***
**Depression**					
No	3,105	686,031	4.53	1.00	1.00
Yes	176	24,783	7.10	1.66 (1.43, 1.94)***	1.45 (1.24, 1.69)***
**Chronic kidney disease**					
No	3,259	708,672	4.60	1.00	1.00
Yes	22	2,142	10.3	2.30 (1.52, 3.50)***	1.49 (0.98, 2.28)
**Obesity**					
No	3,218	700,502	4.59	1.00	1.00
Yes	63	10,312	6.11	1.42 (1.11, 1.82)**	1.27 (0.99, 1.63)
**Cancer**					
No	3,224	704,478	4.58	1.00	1.00
Yes	57	6,336	9.00	2.06 (1.58, 2.67)***	1.54 (1.18, 2.00)**

*Rate^#^, incidence rate, per 1,000 person-years; Crude HR, relative hazard ratio; Adjusted HR^&^: multivariable analysis including age, sex, and comorbidities of diabetes, CAD, depression, chronic kidney disease, obesity, and cancer*.

**p < 0.01, ***p < 0.001.

In the endometriosis and control groups, 764 and 2,517 patients had HZ occurrence, respectively. After adjustments for age and comorbidities, the patients with endometriosis age ≤ 49 years (aHR = 1.17, 95% CI = 1.07–1.28; *p* < 0.001) and 50–64 years (aHR = 1.27, 95% CI = 1.03–1.56; *p* < 0.05) showed a significantly higher HZ risk than the corresponding controls. Among women without any comorbidities, patients with endometriosis were 1.22 times (95% CI = 1.12–1.34; *p* < 0.001) more likely to have HZ than those without endometriosis (Table 3).

**Table 3:** Incidence of herpes zoster by age, sex, and comorbidity and Cox model measured hazards ratio for patients with endometriosis compared those without endometriosis.

	**Endometriosis**							
	**No**			**Yes**				
**Variables**	**Event**	**PY**	**Rate^**#**^**	**Event**	**PY**	**Rate^**#**^**	**Crude HR (95% CI)**	**Adjusted HR^**&**^ (95% CI)**
**Age, years**								
≤ 49	2,104	530,737	3.96	630	133,173	4.73	1.19 (1.09, 1.30)***	1.17 (1.07, 1.28)***
50–64	369	34,987	10.6	117	8,650	13.5	1.28 (1.04, 1.58)*	1.27 (1.03, 1.56)*
65+	44	2,619	16.8	17	647	26.3	1.57 (0.90, 2.75)	1.51 (0.85, 2.66)
**Comorbidity** ^**§**^								
No	2,125	518,602	4.10	620	124,999	4.96	1.21 (1.11, 1.32)***	1.22 (1.12, 1.34)***
Yes	392	49,741	7.88	144	17,471	8.24	1.04 (0.86, 1.26)	1.11 (0.92, 1.35)

*Rate^#^, incidence rate, per 1,000 person-years; Crude HR, relative hazard ratio; Adjusted HR: multivariable analysis including age, sex, and comorbidities of diabetes, CAD, depression, chronic kidney disease, obesity, and cancer*.

§ *Individuals with any comorbidity of diabetes, CAD, depression, and chronic kidney disease, obesity, and cancer were classified into the comorbidity group*.

*p < 0.0, ***p < 0.001.

**Figure 2:**
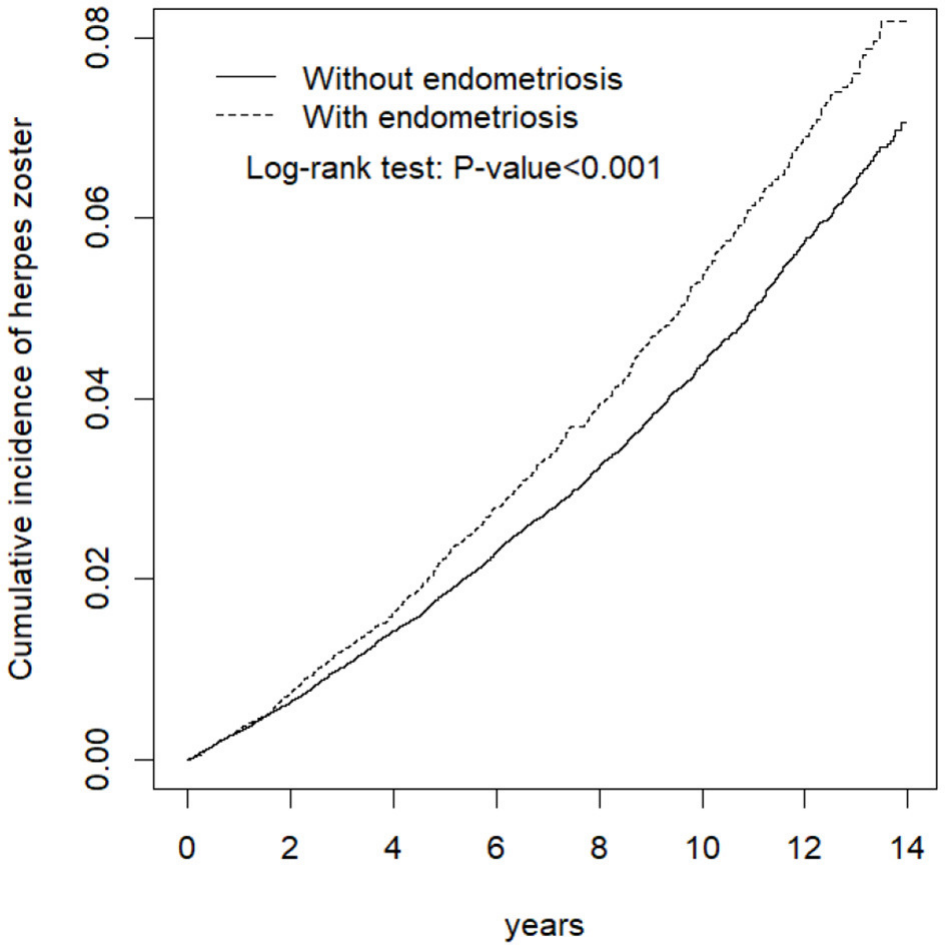
Cumulative incidence comparison of herpes zoster for patients with (dashed line) or without (solid line) endometriosis.

Kaplan-Meier analysis of the cumulative incidence of HZ revealed that a statistically significant difference in HZ occurrence between the endometriosis and control groups (log-rank test *p* < 0.001) (Figure 2).

## Discussion

This is the first population-based study to identify the association between endometriosis and HZ. We found that patients with endometriosis were 1.20 times more likely to have HZ than those without endometriosis.

Chronic pelvic pain (CPP) is a common problem among women with endometriosis. Mowers et al. reported that 21.4% of patients who had undergone laparoscopic or abdominal hysterectomy for CPP found endometriosis (11). The association between endometriosis and depression has also been identified. Friedl et al. investigated depressive syndromes among women with endometriosis by using the Status of Health Questionnaire, Hospital Anxiety and Depression Scale, and Endometriosis Health Profile; and they reported that depressive symptoms were detected in 14.5% of patients with endometriosis (12). However, Roomaney et al. found that 43.1% of women with endometriosis reported moderate to severe symptoms of depression (13). The difference in the rate of depression among women with endometriosis might have been due to the limited number of patients in the study.

Lorençatto et al. found depression among 86 and 38% of women having endometriosis with and without CPP, respectively. They suggested that the prevalence of depression is high among women with endometriosis, particularly in those with CPP (14). A population-based study was conducted by Chen et al. to examine the association between endometriosis and an increased risk of depression development. They found that women with endometriosis were 1.56 times and 1.44 times more likely to develop major depression and any depressive disorders than those without endometriosis (15). Hence, screening of depression may be required for patients with endometriosis.

The association between CPP and depression has been well-defined. Romão et al. evaluated the prevalence of depression by using the Hospital Anxiety and Depression Scale. They found that the prevalence of depression was 40 and 30% among patients with and without CPP, respectively (16). Subsequently, Siqueira-Campos et al. also investigated the prevalence of depression among women with CPP by using the Hospital Anxiety and Depression Scale, and they found depression in 63% of the women with CPP and in 38% of the controls. They concluded that the prevalence of depression was higher in women with CPP (17). Recently, Brooks et al. demonstrated that a higher depression score was associated with a currently higher severity of CPP by using the 21-item Depression, Anxiety and Stress Scale in a sample population of 212 women. It has been determined that the severity of depression is dependent upon the severity of pain (18).

The mechanism of endometriosis-pain-depression was explained by Li et al. They described that endometriosis induced pain sensitization and depression by regulating brain gene expression and electrophysiology. They suggested that the effect of endometriosis on the brain might be the basis for pain sensitization and emotional disorders presented in women with this disease (19). Graziottin et al. found that activated mast cells director inflammatory pathways contributing to CPP and neuropsychiatric diseases, and they suggested that mast cells should be targeted for the multifactorial treatment of pain and depression (20).

Two population-based studies have identified the risk of HZ among patients with depression. Choi et al. reported that the prevalence of HZ was 6.8% among patients with depression and 6.3% among controls (21). Liao et al. found that the incidence of HZ was 4.58 per 1,000 person-years in the depression group and 3.54 per 1,000 person-years in the control group (22). Both of them proposed that patients with depression were 1.1 times more likely to have HZ than patients without depression. Thus, it is evident that depression increases HZ risk.

In the Table 1, the rates of diabetes, CAD, depression and obesity were higher in the case group. And, the patients with diabetes, CAD depression or cancer had a higher rate of HZ occurrence (Table 2). However, among women without any comorbidities, patients with endometriosis had a significantly higher rate of HZ occurrence than those without endometriosis (Table 3). It means that endometriosis represents a high burden in affected women and influences the occurrence of HZ.

In this study, the diagnosis of endometriosis was made from the patient's past history, physical examination, imaging finding or pathologic confirmation through laparoscopy. A previous population-based study from Taiwan, Lee et al. found that only 12% of patients with endometriosis were surgical confirmation (23). However, according to the CNGOF/HAS clinical practice guidelines, the authors mentioned that when imaging is already identified, laparoscopy is not recommended to confirm the diagnosis (24). This population-based study analyzed a subset of the Taiwanese NHIRD, and the selection of patients was based on the diagnostic code. All insurance claims should be submitted to the NHI Administration and reviewed by reimbursement experts through a rigorous review system. In order to make medical resources reasonably used on the insured persons, improve the quality of medical care and avoid unnecessary or inappropriate medical service affecting the reimbursement of necessary and legitimate medical services, the NHI Administration conducts a rigorous review system in accordance with the NHI Insurance Law. According to the Article 63 and 64 of the NHI Insurance Law, medical experts with clinical or relevant experience will be selected to review the insurance claims. The reviews of reimbursement will be focused on the items, quantity and quality of medical service. If the insurer disagrees to pay the expense of prescriptions (such as medication or examination) after reviewing by experts, and the responsibility belongs to this doctor; then, the cost is deducted from the medical expenses declared by the medical institution to which the doctor belongs. In a national study conducted by Lee et al., the authors reported that almost 90% of endometriosis was diagnosed by gynecologists in Taiwan (23). Although diagnosis of endometriosis or HZ may make by different physicians such as gynecologists, dermatologists or general practitioners, but the diagnosis of diseases is considered reliable through the rigorous review system of NHI Administration. However, it still has several limitations. First, self-payment for treatment of diseases was not recorded in the NHIRD. Before December 1, 2018, the NHI coverage of anti-viral medication for HZ was limited to the patients with immune insufficiency, cancers, organ transplantation and the lesions around the genitals or head and neck. The patients with self-paid anti-viral drugs for HZ were not recorded in the NHIRD. Now, anti-viral drugs can be prescribed to the patients with HZ regardless the sites of the lesions and patients' groups. However, the patients with self-paid medical herbs for pain alleviation either endometriosis or HZ were also not recorded in the NHIRD. Therefore, it might lead to the under-diagnosis of endometriosis or HZ. Second, data on the severity of diseases were not available in the NHIRD, which might have influenced the outcomes. Third, lifestyle was also not available. For example, exercise may influence the immunity of body leading to the different outcome of diseases. Moreover, according to previous reports, smoking may also affect the occurrence of HZ and endometriosis (25–27).

Although the retrospective cohort study is usually lower evidence than the randomized controlled trials because a retrospective cohort study is subject to have many unknown or uncontrolled confounding factors and this study only includes a Taiwanese population; however, this paper is the first study to identify the association between endometriosis and HZ risk using a large sample-size on diagnoses, hospitalizations and prescriptions of NHIRD, which may support by a sufficiently statistical ability. Our conclusion suggests that women with endometriosis may have a higher risk of HZ occurrence. The results may provide a reference for the study of population medicine. However, ethnic disparity is needed to determine the association in the further study.

## Conclusion

Taiwanese women with endometriosis may have a higher rate of HZ occurrence. Endometriosis seems to be a high burden for affected women. Therefore, we suggest that clinicians should be aware of HZ among women with endometriosis, although there may be ethnic differences.

## Data Availability Statement

The datasets presented in this article are not readily available because the dataset used in this study is held by the Taiwan Ministry of Health and Welfare (MOHW). The Ministry of Health and Welfare must approve our application to access this data. Any researcher interested in accessing this dataset can submit an application form to the Ministry of Health and Welfare requesting access. Please contact the staff of MOHW (Email: stcarolwu@mohw.gov.tw) for further assistance. Taiwan Ministry of Health and Welfare Address: No.488, Sec. 6, Zhongxiao E. Rd., Nangang Dist., Taipei City 115, Taiwan (R.O.C.). Phone: +886-2-8590-6848. All relevant data are within the paper. Requests to access the datasets should be directed to Email: stcarolwu@mohw.gov.tw.

## Ethics Statement

This study was approved to fulfill the condition for exemption by the Institutional Review Board (IRB) of China Medical University (CMUH104-REC2-115-CR4). Written informed consent for participation was not required for this study in accordance with the national legislation and the institutional requirements.

## Author Contributions

C-YH: conception/design. C-HK: provision of study materials. All authors: collection and/or assembly of data, data analysis and interpretation, manuscript writing, and final approval of manuscript.

## Funding

This study is supported in part by Taiwan Ministry of Health and Welfare Clinical Trial Center (MOHW110-TDU-B-212-124004), China Medical University Hospital (DMR-109-231, DMR-110-089), Tseng-Lien Lin Foundation, Taichung, Taiwan. The funders had no role in the study design, data collection and analysis, the decision to publish, or preparation of the manuscript. No additional external funding was received for this study.

## Conflict of Interest

The authors declare that the research was conducted in the absence of any commercial or financial relationships that could be construed as a potential conflict of interest.

## Publisher's Note

All claims expressed in this article are solely those of the authors and do not necessarily represent those of their affiliated organizations, or those of the publisher, the editors and the reviewers. Any product that may be evaluated in this article, or claim that may be made by its manufacturer, is not guaranteed or endorsed by the publisher.
